# Effect of Tool Shoulder Profile on Grain and Texture Development in the Weld Interface Zone of Friction-Stir-Welded Dissimilar AA2024/AA7075 Joints

**DOI:** 10.3390/ma18020340

**Published:** 2025-01-14

**Authors:** Qi Li, Chenghang Zhang, Jianhong Sun, Haoge Shou

**Affiliations:** 1 College of Intelligent Manufacturing, Huanghuai University, Zhumadian 463000, China; 2 Ningbo Institute of Technology, Beihang University, Ningbo 315800, China; 3 College of Energy Engineering, Huanghuai University, Zhumadian 463000, China; 20222277@huanghuai.edu.cn; 4 Zhumadian Central Hospital Affiliated of Huanghuai University, Huanghuai University, Zhumadian 463000, China

**Keywords:** tool shoulder profile, dissimilar joint, interface zone, grain, texture

## Abstract

Friction-stir-welded dissimilar AA2024/AA7075 joints have an apparent influence on grain and texture development at the weld interface due to differences in physical and chemical properties between the two aluminum alloys. In this work, the effect of tool shoulder profile on grain structure and texture evolution in the center interface zone (CIZ) and bottom interface zone (BIZ) of dissimilar AA2024/AA7075 joints were quantitatively studied by electron back-scattering diffraction (EBSD). The results indicate that abundant fine and coarse equiaxial grains are produced in the CIZ and BIZ of the joints produced with a concentric circle shoulder (CCS) and three-helix shoulder (THS), and the average grain size of the BIZ is lower than that of the CIZ for the same CCS or THS joint. A higher degree of recrystallization occurs in the CIZ of the joint with a CCS than that of the joint with a THS, while a similar degree of recrystallization is presented in the BIZ of the two joints. For the distribution of local misorientation angle between the two sides of the interface in the same CCS or THS joint, the CIZ manifests relatively uniform behavior, while the BIZ presents the characteristics of uneven distribution. Tool shoulder profile has a significant impact on the texture components at the weld interface, which results in different types of shear textures generated in the CIZ and BIZ of the two joints. It is beneficial to make out the microstructural evolution mechanism at the weld interface in dissimilar FSW joints for engineering applications in this study.

## 1. Introduction

With the prominence of global energy and environmental protection issues, the application of lightweight alloy materials in industry is becoming increasingly important [1,2]. Due to their light quality, good thermal conductivity, high strength and other excellent comprehensive performance attributed, aluminum alloys are widely used in industrial production and have become the second largest industrial material after steel materials [3]. As an important non-ferrous material, aluminum alloy is the most diffusely applied non-ferrous material in the field of aerospace. Due to the special demands of work environments in the aerospace field, such as high load, high pressure resistance and high reliability, high requirements are put forward for the combination properties of aluminum alloys [4]. Therefore, aviation aluminum alloys with high strength, low density and high fatigue resistance have been developed [5], mainly including 2xxx- and 7xxx-series aluminum alloys depending on different loads [6]. 2xxx-series aluminum alloy is mainly used in the fuselage skin, lower wing skin and other fuselage structures [7], while 7xxx-series aluminum alloy is mainly used in the aircraft skeleton, wing ribs, bulkhead and other important bearing structures [8].

The joining of dissimilar materials in engineering manufacturing industries is widely applied due to its ability to effectively combine their respective functions. Replacing steel with aluminum in the automotive industry is an effective way to achieve a lightweight structure, which inevitably involves the joining of aluminum to steel [9,10]. The manufacturing of aviation components inevitably involves the welding of these above two types of aluminum alloys [11]. Using a welding method instead of the traditional riveting and mechanical connection methods can simplify the structure and reduce the weight of the body [12]. Since aviation aluminum alloys possess high thermal conductivity and active chemical properties [13], oxide inclusions, cracks, pores and other defects are easily formed during traditional fusion welding, which deteriorates the mechanical performance of aviation aluminum welds and failing to meet the manufacturing requirements of aircraft. As a new type of solid-phase welding technology, friction stir welding (FSW) has great advantages for the welding of aluminum alloys [14] and is more widely used in aviation manufacturing than conventional fusion welding technologies. The melting and solidification processes does not occur in the welded zone during FSW, avoiding various defects generated in the process of fusion welding. As a consequence, FSW can realize welding between different series of aluminum alloys—in particular, welding between 2xxx and 7xxx alloys—on account of its limited production of defects and residual stress compared with traditional welding methods [15,16].

The impacts of welding process parameters (rotation speed, welding speed, tool shoulder profile, etc.) on the microstructure and mechanical performance of dissimilar aluminum alloy FSW joints have been investigated in the literature. Varunraj and Ruban [17] investigated the mechanical properties of dissimilar FSW AA6063/AA7075 joints by altering the tool rotating speed and welding speed, and they found the ideal process window for preparing defect-free joints. Laska et al. [18] revealed that the rotational speed has a prominent impact on the microstructure and mechanical performance of dissimilar FSW AA5083/AA6060 joints. They found that a high hardness value is presented in the weld nugget zone of joints at a high tool rotational speed. Rana et al. [19] investigated the effects of traverse rates on the microstructural evolution and mechanical performance of dissimilar FSW AA7075/AA6061 joints. They found that the grain size in the nugget zone is reduced with an increasing tool traverse rate from 20 to 60 mm/min and that a distinct tensile strength of 251 MPa for dissimilar AA7075/AA6061 joints can be obtained at a traverse rate of 60 mm/min.

In addition, many researchers have conducted the optimality studies of welding process parameters for dissimilar aluminum alloy FSW joints. Rathinasuriyan et al. [20] adjusted the impacts of axial force, rotational speed and traverse feed on the mechanical performance of dissimilar FSW AA2024/AA7075 joints. It can be concluded that the optimal welding condition can be obtained by adjusting the rotational speed, traverse feed and axial force to realize optimal microhardness and tensile strength. This is due to the fact that fine recrystallized grains and adequate material filling can be formed under these condition, avoiding the generation of tunnel defects. Mothilal and Kumar [21] used response surface methodology (RSM) to optimize the processing parameters for dissimilar FSW AA5083-O/AA7075-T6 joints, achieving ideal welding processing parameter combinations and optimized performance can be acquired. Satyanarayana et al. [22] employed Taguchi optimization by selecting the tool tilt angle, rotational speed and feed rate for dissimilar FSW AA8011/AA5052 joints, and the best combinations of processing parameters and mechanical properties were obtained.

The above research shows that insufficient heat input in the process of welding leads to insufficient material flow in the stirring zone, which leads to insufficient material filling and making the joints prone to defects such as holes. Moreover, excessive heat input causes the coarsening of the microstructures including grains and the strengthening phase in the welded zone. Therefore, matched heat input conditions are a prerequisite for preparing high-quality dissimilar joints. However, unmatched thermal input usually causes uneven internal structures in joints; in particular, the weld interface, which is the joining area between two base materials (BMs), tends to be the failure location area when the joint bears the load. Hence, it is necessary to fully understand the microstructural evolution behavior of the weld interface area in dissimilar joints. As one of the main parameters affecting the heat input during FSW, the tool shoulder profile plays a significant role. The impact mechanism of the tool shoulder profile on grain and texture development in the interface area of dissimilar FSW AA2024/AA7075 joints is explored in this investigation so as to provide theoretical support for the practical engineering application of such joints by taking advantage of the electron back-scattering diffraction (EBSD) technique as a function of our preceding investigation [23].

## 2. Experimental Procedure

**Figure 1:**
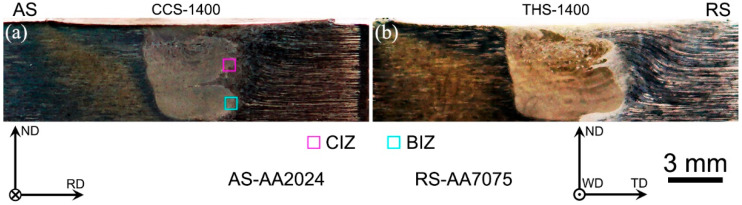
Cross-section appearances of dissimilar AA2024/AA7075 FSW joints: (**a**) CCS joint; (**b**) THS joint.

The two selected BMs were AA2024-T351 and AA7075-T651 alloy sheets with dimensions of 300 mm × 40 mm × 5 mm. The corresponding chemical compositions and mechanical properties are exhibited in Table 1. Two BMs were firmly attached to the FSW device for butt welding. AA2024-T351 and AA7075-T651 sheets were placed on the advancing side (AS) and retreating side (RS), respectively. H13 hardened steel was used as the welding tool, and two types of tool shoulder profiles (concentric circle shoulder (CCS) an d three-helix shoulder (THS)) were employed [23]. Their detailed sizes are exhibited in Table 2. During FSW, the tool rotational speed, welding speed, tool tilting angle and tool plunge depth were maintained at 1400 rpm, 120 mm/min, 2.5° and 0.1 mm, respectively, based on the ideal welding process window obtained in previous work [24]. The position relationships of the rolling direction (RD) of the two BMs, the tool welding direction (WD) and the transverse direction (TD) of the joints are drawn in Figure 1. The joint cross section is perpendicular to the WD and was applied to perform EBSD characterization. The EBSD test with was carried out by utilizing a field-emission scanning electron microscope (FESEM, TESCAN MIRA 3, Brno, Czech) that was operated at 20 kV and equipped with an HKL-EBSD system (Oxford, UK, the step size was set to be 0.3 µm). Channel 5 software (2019 v5.12) was used for data processing.

## 3. Results and Discussion

As shown by the cross-sectional structural analysis presented in Figure 1 [23], defect-free dissimilar AA2024/AA7075 FSW joints were successfully prepared by utilizing CCS and THS tools. In addition, it was found that the welded zone presents the characteristics of a basin through a morphology analysis. The welded zone is composed of two BMs and presents significant interface characteristics. Therefore, the local microstructure of the weld interface zone in the dissimilar joints is analyzed in this work. It is divided into two local regions by the EBSD characterization technique, namely the center interface zone (CIZ) and the bottom interface zone (BIZ).

**Figure 2:**
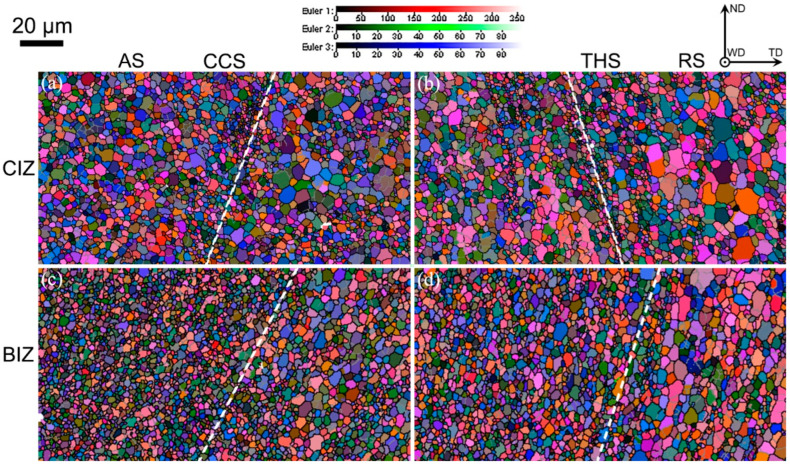
Euler angle diagrams (Euler angles 1, 2 and 3 represent the angle (^o^) of rotation around ND, WD and TD, respectively) in the welded zone: (**a**) CCS-CIZ; (**b**) THS-CIZ; (**c**) CCS-BIZ; (**d**) THS-BIZ.

As exhibited in Figure 2 [23], an uneven grain structure is found in the interfacial region of dissimilar CCS and THS joints. Specifically, abundant equiaxial grains are generated in the CIZ and BIZ of dissimilar CCS and THS joints, which are mainly composed of fine and coarse grains. Fine equiaxed grains are mainly distributed in the interface regions and the region towards the AS, while coarse grains are primarily distributed in the welded area towards the RS. According to statistical analysis [23], the average grain size in the CIZ and BIZ of a CCS joint is 2.25 ± 1.33 μm and 1.61 ± 0.83 μm, respectively, while the average grain size in the CIZ and BIZ of the THS joint is 2.23 ± 1.46 μm and 1.86 ± 1.07 μm, respectively. It can be concluded that the CIZ and BIZ of the two joints possess almost the same average grain size, while a relatively uniform grain structure is found in the welded zone of the joint with a CCS in comparison with the joint with THS. This is owing to the fact that the thermal cycle produced by the CCS is more uniform than the joint with THS, which causes more adequate material flow in the welded zone of the CCS joint than that of the THS joint. Moreover, for the same CCS or THS joint, it can be found that the fine-grain count in the BIZ is obviously higher than that in the CIZ and the average grain size of the BIZ is obviously inferior to that of the CIZ on account of the fact that heat dissipation rate in the BIZ is faster than that in the CIZ, since the BIZ is close to the substrate, which causes a higher cooling rate in the BIZ than that in the CIZ, resulting in the production of fine equiaxial grains. Cho et al. [25] also found that the grain size exhibits a gradient behavior along the thickness direction of the welded zone in dissimilar FSW AA5083/AA6082 joints.

**Figure 3:**
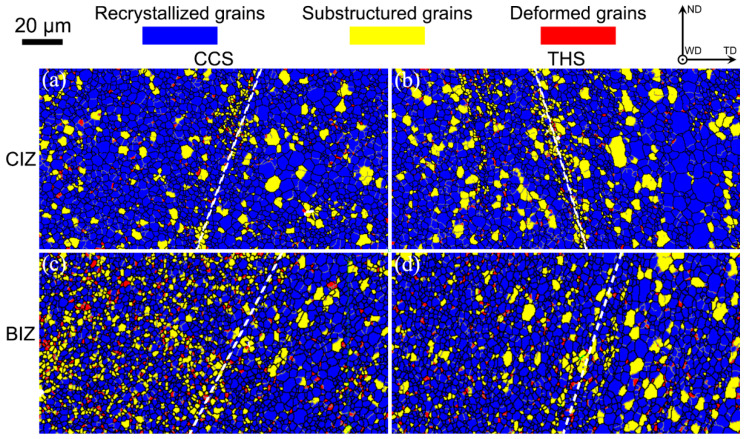
Analysis maps of the grain recrystallization degree in the welded zone: (**a**) CCS-CIZ; (**b**) THS-CIZ; (**c**) CCS-BIZ; (**d**) THS-BIZ.

**Figure 4:**
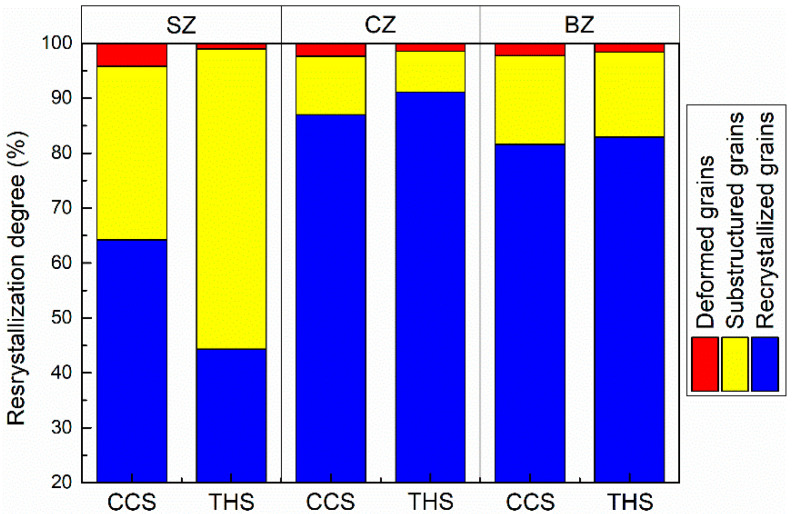
Statistical results of the grain recrystallization degree in the welded zone of the dissimilar joints shown in Figure 3.

The grain recrystallization degree in the weld interface areas of dissimilar CCS and THS joints is demonstrated in Figure 3, the calculated recrystallized, substructured and deformed grains are summarized in Figure 4. It can be observed from Figure 3 that some substructured grains are distributed near the interface area, including the CIZ and BIZ of the two joints, which results from the fact that the two BMs meet and mix with each other in the interface zone and material flow near the interface zone is not sufficient compared to other local weld zones. It can be found that the fraction of the recrystallized grains in the CIZ of the CCS and THS joints is 84.5% and 73.49%, respectively. More recrystallized grains are formed in the CIZ of the joint with a CCS than that of the joint with a THS, while more substructured grains are generated in the CIZ of the THS joint than that of the CCS joint. Since relatively sufficient material flow occurs in the CIZ of the joint with a CCS in comparison with to the joint with a THS, a higher degree of recrystallization occurs in the CIZ of the joint with CCS than that of the joint with a THS. In addition, the proportion of recrystallized, substructured and deformed grains in the BIZ of the CCS joint is 73.49%, 19.87% and 6.64%, respectively. The proportion of recrystallized, substructured and deformed grains in the BIZ of the THS joint is 76.29%, 18.87% and 4.84%, respectively. It can be concluded that similar fractions of recrystallized, substructured and deformed grains are produced in the BIZ of the two joints, which proves that similar behavior of material flow occurs in the BIZ of the CCS and THS joints.

**Figure 5:**
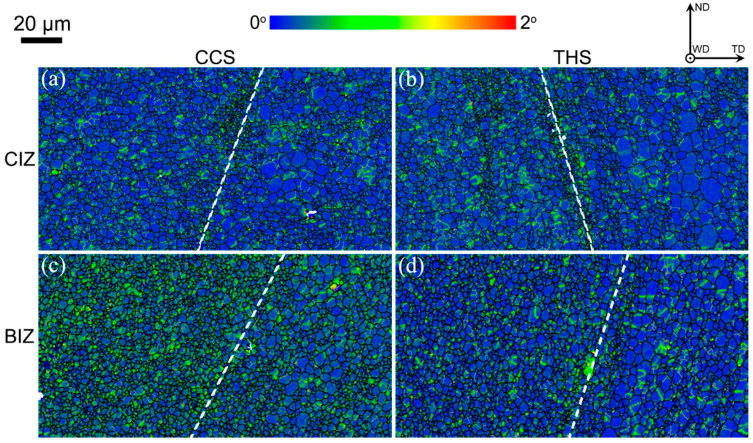
Distribution images of local misorientation in the welded zone: (**a**) CCS-CIZ; (**b**) THS-CIZ; (**c**) CCS-BIZ; (**d**) THS-BIZ.

**Figure 6:**
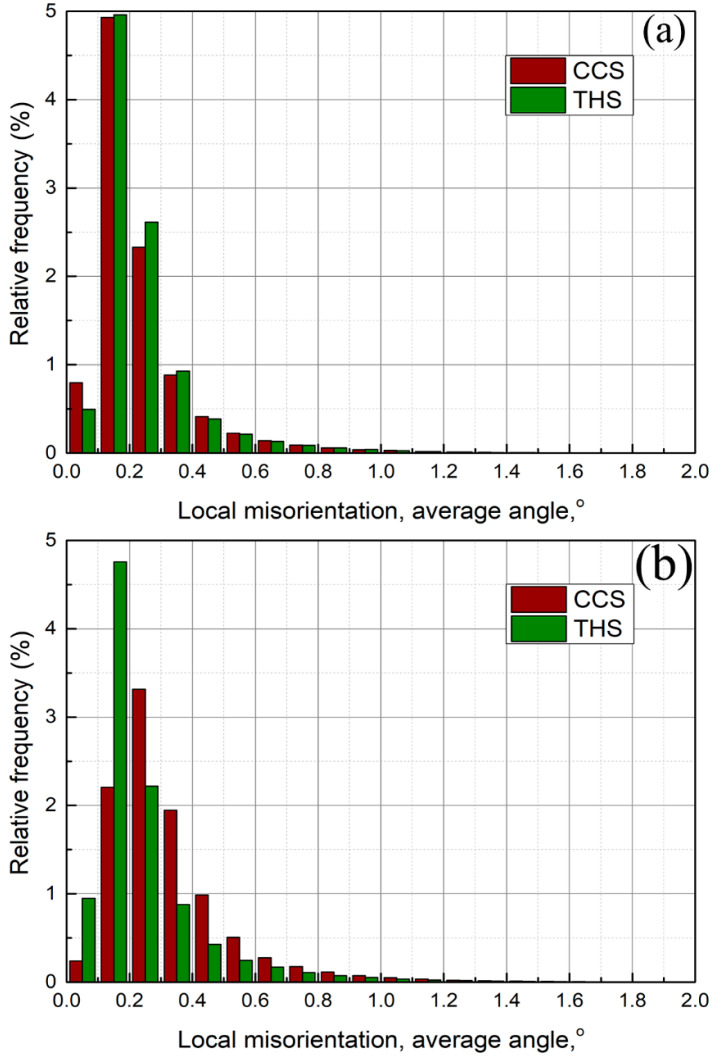
Statistical results of local misorientation in the welded zone shown in Figure 5: (**a**) CIZ; (**b**) BIZ (relative frequency is described as the occurrence frequency of a local misorientation within the set local misorientation range).

Distribution images of local misorientation in the welded zone are demonstrated in Figure 5, which exhibits the intragranular information of the local orientation gradient. It can be found from Figure 5 that the local misorientation angle at the interface line of the CIZ and BIZ is larger than that on both sides (AS and RS), indicating that there is a high dislocation density at the interface, which results from insufficient material flow. This is consistent with the consequence of high fraction of substructural grains at the interface shown in Figure 3. Statistical results of local misorientation in the welded zone of the dissimilar joints shown in Figure 5 are displayed in Figure 6. For the CCS and THS joints, it can be obviously observed that the local misorientation angle distribution between the two sides of the CIZ and the BIZ presents uniform and uneven distribution behavior, respectively. Furthermore, the local misorientation angle in the CIZ of the joint with a CCS is inferior to that of the joint with a THS. This is mainly because material flow in the CIZ is more adequate than that in the BIZ for the two joints, resulting in relatively uniform distribution of the local misorientation angle in the CIZ. However, a fast cooling rate and limited plastic deformation cause insufficient material flow in the BIZ, leading to uneven distribution of the local misorientation angle.

**Figure 7:**
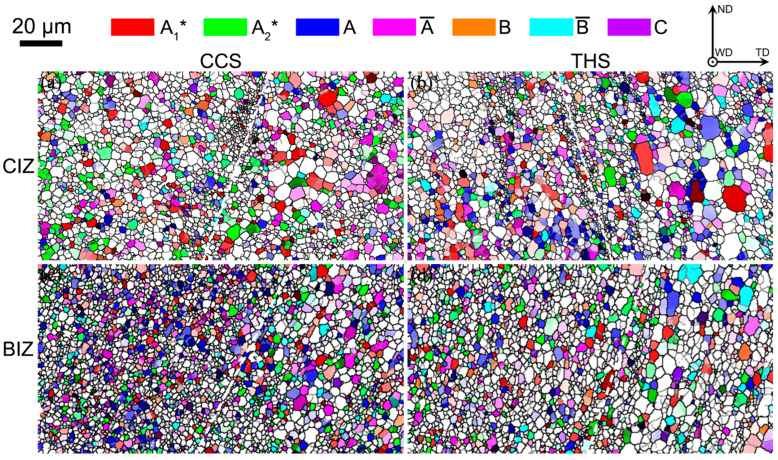
Distribution images of shear texture components in the welded zone: (**a**) CCS-CIZ; (**b**) THS-CIZ; (**c**) CCS-BIZ; (**d**) THS-BIZ.

**Figure 8:**
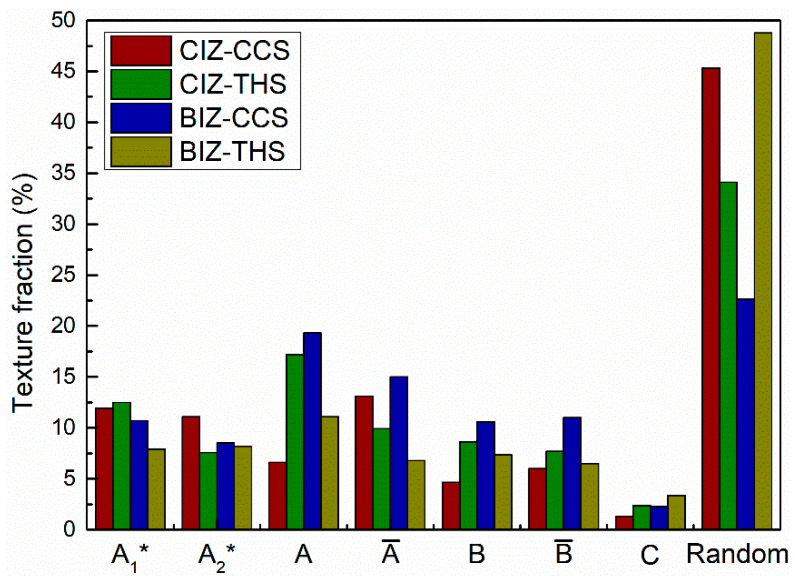
Statistical results of shear textures in the welded zone of the dissimilar joints shown in Figure 7.

During FSW, the BM that is in contact with the stirring tool in the weld is subjected to the friction and extrusion of the stirring tool, which results in the shear strain state of the BM, ultimately forming shear texture in the weld. There are seven types of texture components for face-centered cubic (FCC) metal after shear deformation: $\left\{111\right\}\langle \bar{1}\bar{2}2\rangle \;A_{1}^{*}$, $\left\{111\right\}\langle 11\bar{2}\rangle \;A_{2}^{*}$, $\left\{1\bar{1}1\right\}\langle 110\rangle \;A$, $\left\{\bar{1}1\bar{1}\right\}\langle \bar{1}\bar{1}0\rangle \;\bar{A}$, $\left\{1\bar{1}2\right\}\langle \bar{1}1\bar{2}\rangle \;B$, $\left\{\bar{1}1\bar{2}\right\}\langle \bar{1}\bar{1}0\rangle \;\bar{B}$ and $\left\{001\right\}\langle 110\rangle \;C$ [26,27]. Figure 7 shows distribution images of shear texture components in the interface areas of dissimilar CCS and THS joints and the statistic results are shown in Figure 8. It can be found that multiple shear texture components are formed in the CIZ and BIZ of these two joints. In the CIZ, more shear textures are formed in the THS joint than in the CCS joint. $A_{1}^{*}$, $A_{2}^{*}$ and $\bar{A}$ are the predominant components in the CIZ of the CCS joint, while $A_{1}^{*}$, A, $\bar{A}$ and B are predominant in the CIZ of the joint with a THS. In the BIZ, more shear texture components are formed in the CCS joint than in the THS joint. The shear texture components in the BIZ of the CCS joint are mainly composed of $A_{1}^{*}$, $A_{2}^{*}$, A, $\bar{A}$, B and $\bar{B}$, while the shear textures in the BIZ of the joint with a THS mainly consist of $A_{1}^{*}$, $A_{2}^{*}$, A and B components. Based on the above analysis results, the shoulder tool profile has a significant impact on the texture in the weld interface region. The influence of the two tools on the formation of shear textures in the CIZ and BIZ of dissimilar joints is complicated. The impact of the THS tool on the generation of shear texture components in the CIZ is significant compared to the CCS tool, while CCS tool has an apparent impact on the production of shear textures in the BIZ compared to the THS tool. Malakar et al. [28] found similar results, and they concluded that shear texture components and the strength of the friction stir processing aluminum samples are significantly impacted by the processing parameters, summarizing major shear textures in the welded zone during FSW of aluminum alloys. Wang et al. [29] also concluded that $A_{1}^{*}$ and C components are formed in the welded zone of dissimilar FSW AA5052-O/AA6061-T6 joints.

## 4. Conclusions

In the present investigation, grain and texture development in the interface zone of dissimilar aluminum alloy joints used in aerospace were quantitatively studied by employing different tool shoulder profiles. The specific conclusions are as follows:

1. Abundant fine and coarse equiaxial grains are produced in the CIZ and BIZ of dissimilar joints by CCS and THS. Fine equiaxed grains are primarily generated in the interface regions and the region towards the AS, while coarse grains are primarily distributed in the welded area towards the RS.
2. Since a higher cooling rate occurs in the BIZ, which is close to the substrate, than in the CIZ, the average grain size of the BIZ is lower than that of the CIZ for the same CCS or THS joint.
3. Due to sufficient material flow, a higher degree of recrystallization occurs in the CIZ of the joint with CCS than that of the joint with a THS. Similar fractions of recrystallized, substructured and deformed grains are produced in the BIZ for joints with a CCS or THS.
4. The distribution of the local misorientation angle between the two sides in the CIZ and BIZ for CCS and THS joints presents uniform and uneven behavior, respectively, mainly influenced by the degree of material mixing.
5. Multiple shear texture components are generated in the CIZ and BIZ of CCS and THS joints. More shear textures are engendered in the CIZ of the THS joint than that of the CCS joint, while fewer shear textures are engendered in the BIZ of the THS joint than that of the CCS joint.

Since the welded zone in dissimilar joints mainly experiences asymmetric heat input and shear deformation during FSW, an uneven microstructure and texture are engendered in the local welding zone. Therefore, it is necessary to conduct further research on the action mechanism of temperature and strain rate generated on the grain structure, texture type and strength in the welded zone of dissimilar joints in the process of FSW for engineering applications.

## Figures and Tables

**Table 1:** Chemical compositions of the two BMs (wt.%).

BM	Al	Zn	Mg	Cu	Mn	Ti	Cr	Si	Fe	Yield Strength	Tensile Strength	Elongation
AA2024-T351	Bal.	0.03	1.4	4.5	0.6	0.02	0.01	0.05	0.17	360 MPa	470 MPa	20.3%
AA7075-T651	Bal.	5.8	2.4	1.7	0.04	0.03	0.2	0.05	0.19	476 MPa	555 MPa	11.4%

**Table 2:** Tool dimensions of the CCS and THS tools (mm).

Tool	Shoulder Diameter	Pin-Tip Diameter	Pin Length
CCS	15	3.76	5
THS	18	4.52	5

## Data Availability

The original contributions presented in this study are included in the article. Further inquiries can be directed to the corresponding authors.
